# Loss of RE-1 silencing transcription factor accelerates exocrine damage from pancreatic injury

**DOI:** 10.1038/s41419-020-2269-7

**Published:** 2020-02-20

**Authors:** Julie K. Bray, Ola A. Elgamal, Jinmai Jiang, Lais S. Wright, Dhruvitkumar S. Sutaria, Mohamed Badawi, Madeline G. Borcyk, Xiuli Liu, Kristianna M. Fredenburg, Martha L. Campbell-Thompson, Thomas D. Schmittgen

**Affiliations:** 1 Department of Pathology, Immunology, and Laboratory Medicine, College of Medicine, University of Florida, Gainesville, FL USA; 2 Department of Pharmaceutics, College of Pharmacy, University of Florida, Gainesville, FL USA; 3 Division of Pharmaceutics and Pharmaceutical Chemistry, College of Pharmacy, Ohio State University, Columbus, OH USA

**Keywords:** Transcriptional regulatory elements, Transdifferentiation, Acute inflammation, Chronic inflammation

## Abstract

Regulation of pancreas plasticity is critical for preventing injury and promoting regeneration upon tissue damage. The intricate process of pancreatic differentiation is governed by an orchestrated network of positive and negative transcription factors for appropriate gene expression. While the transcriptional repressor REST is well characterized as a silencer of neuronal genes in non-neuronal cells, the role of REST in regulating exocrine pancreas cell identity remains largely unexplored. *Rest* expression is increased upon injury in the mouse pancreas, such as induced acute and chronic pancreatitis and ductal adenocarcinoma. At the cellular level, *Rest* expression is lower in mature acinar cells compared with pancreas progenitor and ductal cells. To investigate the role of REST activity in pancreatic transdifferentiation and homeostasis, we developed a novel mouse model (Cre/REST^fl/fl^) with conditional knockout (KO) of *Rest* expression within pancreas cells. The high Cre-mediated excision efficiency of *Rest* exon two KO caused decreased *Rest* expression and activity within the pancreas. Short-term organoid cultures of pancreatic acini to undergo acinar-to-ductal metaplasia (ADM) showed that loss of REST impedes induced ADM, while overexpression of REST increases ADM. Interestingly, REST ablation accelerated acute pancreatitis in mice treated with the cholecystokinin analog caerulein, as indicated by cellular morphology, elevated serum amylase levels and pancreatic edema. Furthermore, Cre/REST^fl/fl^ mice were more sensitive to acute pancreatitis injury and displayed augmented tissue damage and cellular lesions. These results suggest REST has a novel protective role against pancreatic tissue damage by acting as a regulator of exocrine cell identity.

## Introduction

Pancreas functions are vital for the proper digestion of food and regulation of blood glucose levels. Digestive enzymes and bicarbonate are produced from the exocrine cells (acinar and ductal cells, respectively), and hormones are produced from the islet neuroendocrine cells. Mature pancreatic cells have a remarkable phenotypic plasticity to transdifferentiate among the different pancreatic cell types and properties (reviewed in ref. ^1^). While this fluid-identity provides protection from environmental changes and recovery from injury, it also lays the foundation for numerous clinical complications when proper regulation of plasticity is lost^2^.

Understanding the molecular mechanisms governing pancreatic cell differentiation has been the focus of numerous therapeutic research fields, such as type 1 diabetes, chronic pancreatitis, and pancreatic cancer^3–6^. One master regulator of directing progenitor maturation is the RE-1 silencing transcription factor (REST)^7^. REST, also known as neuron restrictive silencing factor (NRSF), has been most characterized as a seminal negative regulator that represses transcription of neuronal-related genes^8^. The regulation of REST is a highly intricate and tightly controlled process based on spatial and extracellular cues^9^. The appropriate downregulation of REST orchestrates neuronal differentiation that is required for neurogenesis^10,11^ and includes pancreatic neuroendocrine differentiation^12–14^. Known REST targets encompass a variety of genes involved in synaptic formation, cellular trafficking, ion channels, cytoskeletal components, neurotransmitter receptors, and more^15,16^. Since its discovery^17,18^, the role of REST has expanded beyond neuronal development. There are now almost 2000 genes with predicted REST binding sites^15,16,19^. Furthermore, up to 90% of REST target genes are tissue and cell-type specific^20,21^.

While REST has been investigated in the context of pancreatic endocrine development^22,23^, little is known about REST’s role in exocrine cell plasticity. Acinar cells comprise the majority of pancreas mass and have a high capacity to dedifferentiate and transdifferentiate into ductal-like cells in response to injury, such as inflammation or mutational burden^24–26^. This change in cell identity is called acinar-to-ductal metaplasia (ADM), which makes the exocrine cells more refractory to insults until homeostasis is re-established^27^. During a state of chronic injury, regulation of proper cell identity is lost, and ADM lesions can progress into pancreatic cancer (reviewed in ref. ^28^). While studies have broadly reported that REST is expressed in both acinar and ductal cell types^29^, a more thorough investigation is warranted to identify exocrine pancreas cell-specific differences of REST.

To investigate the phenotypic role of REST in maintaining exocrine pancreas homeostasis, we developed a novel mouse model with REST conditionally knocked out (KO) in the exocrine pancreas. Results show that loss of REST increased zymogen granule production within the acinar cells. Induced transformation of REST KO acinar explants in vitro showed an impeded ability to transform into ductular cells. Furthermore, abrogation of REST activity in mice increased sensitivity to induced acute pancreatitis injury, suggesting that REST plays a protective function against exocrine pancreatic damage. Our findings introduce a novel role of REST for maintaining exocrine pancreas homeostasis and regulation of acinar plasticity in response to injury.

## Materials and methods

### Animals

All animal work was done in compliance to a protocol approved by the University of Florida Institutional Animal Care and Use Committee. p48-Cre^+/−^ mice (also known as Ptf1a-Cre^+/−^ mice) from a C57BL/6J background were a generous gift from Dr. Paul Grippo. REST^floxP/floxP^ mice came from a C57BL/6J background and were a generous gift from Dr. Jenny Hsieh^30^. Conditional REST knockout mice (p48-Cre^+/−^/REST^fl/fl^) mice were bred in-house by crossing p48-Cre^+/−^/REST^fl/fl^ females with REST^fl/fl^ males. Pup tail snips were digested in DirectPCR Lysis Reagent (Qiagen) supplemented with 20 mg/ml Proteinase K (Invitrogen) overnight at 55 °C then heat-treated at 85 °C for 45 min. Crude DNA was genotyped for the Cre Recombinase transgene and floxed *Rest* by PCR using LongAmp *Taq* DNA Polymerase (BioLabs). PCR products were resolved on a 1.2% agarose gel for 45 min at 120 V, resulting in a band at 450 base pairs for Cre transgene positive pups, a band at 550 base pairs for WT *Rest* allele, and a band at 700 base pairs for floxed *Rest* allele. PCR primer sequences are as follows: Cre Sense Primer (P1): 5′-CCTGGA AAATGCTTCTGTCCG-3′; Cre antisense primer (P2): 5′-CAGGGTGTTATAAGCAATCCC-3′; REST sense primer (P3): 5′-ACAGGATCTCTAGGAGCTCAGACTGG-3′; REST antisense primer (P4): 5′-CCAGGGTTCAGTTCTCTACACCCAC-3′. Mice were randomized to experimental groups according to age and sex so that near equal representation were in both groups.

### Genomic pancreas DNA isolation and sequencing

To confirm Cre-mediated excision of pancreatic DNA, a subset of 1 to 2-month-old mice were euthanized and their pancreases excised before flash freezing. Tissue was crushed using a chilled stainless steel mortar and pestle then lysed in digestion buffer supplemented with 20 mg/ml Proteinase K (Invitrogen) using a Genomic DNA Isolation Kit (Norgen Biotek Corp). PCR amplification was performed on 250 ng genomic DNA using the LongAmp *Taq* DNA polymerase kit (New England Biolabs) and primers flanking the targeted *Rest* exon two (sense primer (P5): 5′-GAGCCGTTTCCTGTGATGGCATTC-3′; antisense primer (P4): 5′-CCAGGGTTCAGTTCTCTACACCCAC-3′). DNA was amplified for 30 cycles (94 °C for 30 s; 64 °C for 1 min; 65 °C for 2.5 min), and PCR products were electrophoresed on a 0.8% agarose gel at 80 V for imaging. Sanger Sequencing of PCR products were performed by Eton Biosciences using primers P4 and P5.

### Western blot

Pancreas from 1 to 3-month-old mice were flash frozen and crushed using a cooled stainless steel mortar and pestle. Tissue was lysed in Cell Lytic MT Cell Lysis buffer (Sigma), 5 mM EDTA (Thermo), and Halt Protease and Phosphatase Inhibitor Cocktail (Thermo) then homogenized for 15 s. Samples were centrifuged for 15 min at 14,000 × *g* at 4 °C, and supernatant protein concentration was quantified using the BCA Assay (Thermo). Protein was prepared using Bolt LDS Sample Buffer (Novex) and Bolt Sample Reducing Buffer (Novex) then heat denatured at 70 °C for 10 min. Protein samples (15–44 μg) were separated on a 4–12% Bis-Tris Plus Gel (Invitrogen) (80 V for 20 min; 120 V for 60 min) then transferred to a PDVF membrane using Trans-Blot Turbo Transfer System (BioRad). The membrane was blocked in 5% milk solution for 1 h at room temperature and incubated in primary antibody overnight at 4 °C. Membranes were incubated in goat anti-rabbit-HRP or goat anti-rabbit-HRP antibody for one hour at room temperature and imaged using densitometry methods (Amersham ECL Detection (GE Healthcare); Amersham Imager 680 (GE Healthcare)). The following primary antibodies were used: anti-amylase (1:1000, Cell Signaling, 3796), anti-Snap25 (1 μg/ml, Abcam, ab41455, anti-REST (1:1000, Abcam, ab202962), anti-REST (1:500, Millipore, 07-579), anti-REST (1:1000, Abcam, ab21635), anti-REST (1:500, Hsieh514^31^), anti-REST (1:500, Aviva, ARP32086_P050), anti-REST (1:1000, Proteintech 2242–1-AP), anti-β-actin (1:1000, Cell Signaling, 4970S), and anti-GAPDH (1:1000, Cell Signaling, 5174S).

### Quantitative PCR

RNA was harvested and isolated from mouse pancreas according to a previously established protocol^32^ using the miRNeasy Mini Kit (Qiagen). RNA samples had a RIN value of 7.0 or greater (Agilent 2100 Bioanalyzer). Reverse Transcription PCR was done using M-MLV Reverse Transcriptase (Thermo Fisher). Quantitative PCR was performed using EXPRESS SYBR GreenER (Invitrogen) or TaqMan (Applied Biosystems), and fluorescence measurements were collected using the Applied Biosystems, QuantStudio 7 Flex. Primer sequences are listed in Supplemental Table 1. In-house primers were designed using Primer Express (Applied Biosystems). Data were analyzed as gene expression using the 2^−∆CT^ method relative to 18S rRNA or as fold change in gene expression using the 2^−∆∆CT^ method. Statistical analysis was performed on either ∆Ct or fold change data.

### Fasted blood glucose and glucose tolerance test

For determining glucose tolerance, 2 to 4-month-old male and female mice were transferred to clean cages for an overnight fast before blood glucose levels were determined using a glucometer^33^. Mice were injected intraperitoneally with 1 mg glucose/g body weight, and blood glucose levels measured at 0, 30, 60, 90, and 120 min. Blood glucose clearance was quantified as area under the curve using the trapezoidal method.

### Organoid culture for ADM

Acinar-to-ductal metaplasia from mouse acini was induced in vitro using modified Matrigel organoid-based culture techniques described previously^34^. Briefly, 6–8-week-old mouse pancreas was minced to 1–3 mm sections and digested in 0.2 mg/ml Collagenase P (Roche) for 25 min at 37 °C. Clusters were filtered through a 500 µm cell strainer (pluriSelect) and resuspended in Waymouth media supplemented with 10% FBS, 1% Pen Strep, 100 µg/ml trypsin inhibitor (Sigma), and 20 µg/ml dexamethasone (Sigma). For REST KO studies, cells were plated in 50% Matrigel culture (Corning) on a 96-well plate at 100 µl/well. For virus infection studies, primary mouse acinar cells were infected with mouse control expressing adenovirus (abm, Cat. No. m009) or mouse *Rest* adenovirus (abm, Cat. No. 213793A). For adenovirus transduction, equal volumes of cell suspension were added to 5 µl of either control or *Rest* stock mouse adenovirus (10^6^ pfu/ml), incubated at 37 °C and 5% CO_2_ for 1.5 h with gentle rocking every 10 min. Two parts of Matrigel (Corning) was added to one part of virus transduced cell suspension then plated in a 24-well collagen coated plate. The collagen coat was made using collagen I, rat tail (Thermo Fisher Scientific) at 2.6 mg/ml. For all organoid experiments, gelated Matrigel cultures were supplemented with prepared Waymouth media and refreshed every 1–2 days. Cultures were maintained at 37 °C in 5% CO_2_. Microscope slide-grids (Sigma) beneath the plate were used to manually count number of clusters at designated time points. Ductal-like clusters and acinar clusters were defined as 3–10 co-adherent cells, and % ADM was calculated as (# ducts/# total clusters)/well. RNA was isolated at day 1 and day 4 of plating by lysing the Matrigel culture in cold Trizol LS (Ambion).

### Acute pancreatitis model

Two to three-month-old mice were administered eight hourly intraperitoneal injections per day of saline control or 100 µg/kg caerulein (Bachem) over 2 consecutive days for 16 injections total. Mice were euthanized at day 2, 4, or 7 after injections, and the pancreas and blood were collected for analysis.

### Pancreatic histology

Mice were euthanized by isoflurane followed by cervical dislocation. The pancreas was excised and immediately fixed in 10% neutral buffered formalin or 4% formaldehyde. Four-micrometer pancreatic tissue sections were stained with H&E, and histopathology was evaluated by a pathologist blinded to treatment and genotype. Percentage ADM was estimated as the ratio of tissue area containing ADM lesions relative to whole tissue area. Total necrosis score was the product of intensity score (0 = none, 1 = mild, 2 = moderate, 3 = severe) and area of necrosis (0 = none, 1 < 25%, 2 = 25–50%, 3 = 50–75%, 4 > 75%). Total inflammation score was the product of intensity score (0 = none, 1 = mild, 2 = moderate, 3 = severe) and area of inflammation (0 = none, 1 < 25%, 2 = 25–50%, 3 = 50–75%, 4 > 75%). Atrophy score and edema score were defined according to percent area (0 = none, 1 < 25%, 2 = 25–50%, 3 = 50–75%, 4 > 75%). Total histology score was the sum of total necrosis, total inflammation, and edema score.

### Serum amylase levels

Mice were euthanized following isoflurane administration, and blood was collected by cardiac drainage using a needle-less 1-ml syringe to minimize hemolysis. Blood was incubated at room temperature for 25 min to allow coagulation. Samples were centrifuged at 1600 × *g* for 10 min at 4 °C, and the resulting serum was aliquoted and frozen. Aliquots were assayed by the Ohio State University Comparative Pathology and Mouse Phenotyping Shared Resource for amylase activity using the chromogenic method at 408/647 nm wavelength (Vet Axcel Chemistry Analyzer, Wasserman).

### Pancreatic edema

The entire pancreas was excised, and the wet weight was recorded. Tissue was incubated at 85 °C for 24 h, and the dry weight was measured. Percent water content was calculated as ((wet weight − dry weight)/wet weight) × 100.

### Transmission electron microscopy

Pancreas was excised and immediately preserved in Tyrode’s buffer containing 4% paraformaldehyde and 1% glutaraldehyde (pH 7.4), minced into 1-mm sections, and stored overnight at 4 °C. Samples were processed by the University of Florida College of Medicine Electron Microscopy Core Facility according to previously used protocol^35^.

### Immunofluorescence

Mouse pancreas was fixed in 10% neutral buffered formalin and processed to paraffin sections. Sections were deparaffinization using xylene and rehydrated to water using previously reported methods^36^. Antigen retrieval was performed by heating for 20 min in EDTA buffer using a steamer then cooled to room temperature for 20 min. Samples were blocked in 10% goat serum (Vector) for 20 min. Tissue sections were incubated in rabbit anti-E-cadherin (1:300, GeneTex, GTX100443) overnight at 4 °C followed by detection using Alexa Fluor 555-conjugated goat anti-rabbit antibody for 20 min. For amylase and cytokeratin19 staining, sections were deparaffinized and antigen retrieval performed by citrate buffer (AR6) using a microwave for 90 s at full power followed by 15 min at 20% of full power and cool down 30 min. Sections were incubated with TRIS buffer containing 10% normal goat serum for 10 min. Tissue sections were incubated in rabbit anti-amylase (1:2000, Sigma A8273) for 1 h followed by Opal Polymer HRP Ms + Rb for 10 min then tertiary Opal 520 (1:500) for 10 min. Slides were blocked in 10% normal goat serum for 20 min and incubated in rabbit anti-cytokeratin19 (1:100, Abcam ab52625) for 1 h followed by Alexa Fluor 555 for 1 h. Slides were counterstained with DAPI (1 μg/ml, Fisher Scientific, 62248) or Hoechst S769121 (0.5 μg/ml, Abcam, ab138903) stain and imaged using a Zeiss Axioskop 2 Plus Fluorescence microscope.

### Gene set enrichment analysis

Gene set enrichment analysis (GSEA) was performed using the GSEA software at the Broad Institute^37^. The gene expression of eight normal human pancreas and 14 pancreatic ductal adenocarcinoma tissues (GSE71989) was used as the expression dataset. For the gene set database, we used 971 REST target genes^38^, as well as a much smaller set of 24 REST target genes that were indicative of an aggressive subset of breast cancers^39^. Another GSEA was performed using the gene sets to 572 transcription factors and transcription factor binding sites. For all analyses, the permutation type was set to phenotype and 1000 permutations were run.

### Statistical analysis

Statistical analysis was performed using GraphPad Prism 7 (San Diego, CA) and Sigma Plot 13.0 (Systat Software, Chicago, IL). No animals were excluded from the analysis as outliers. Two-sided *p*-values for two-sample comparisons were calculated using the Student’s *t*-test or Mann–Whitney rank sum test. For multiple comparisons, one-way or two-way ANOVA was used, and Holm-Sidak method was applied for post hoc multiple comparison adjustments. Data were transformed as needed to obtain normality and equal variance for experiments with *n* > 5. Expression values of Gene Expression Omnibus datasets were calculated by the anti-log2 of the signal intensity.

## Results

### REST gene expression is increased during pancreatic injury and acinar de-differentiation

**Fig. 1:**
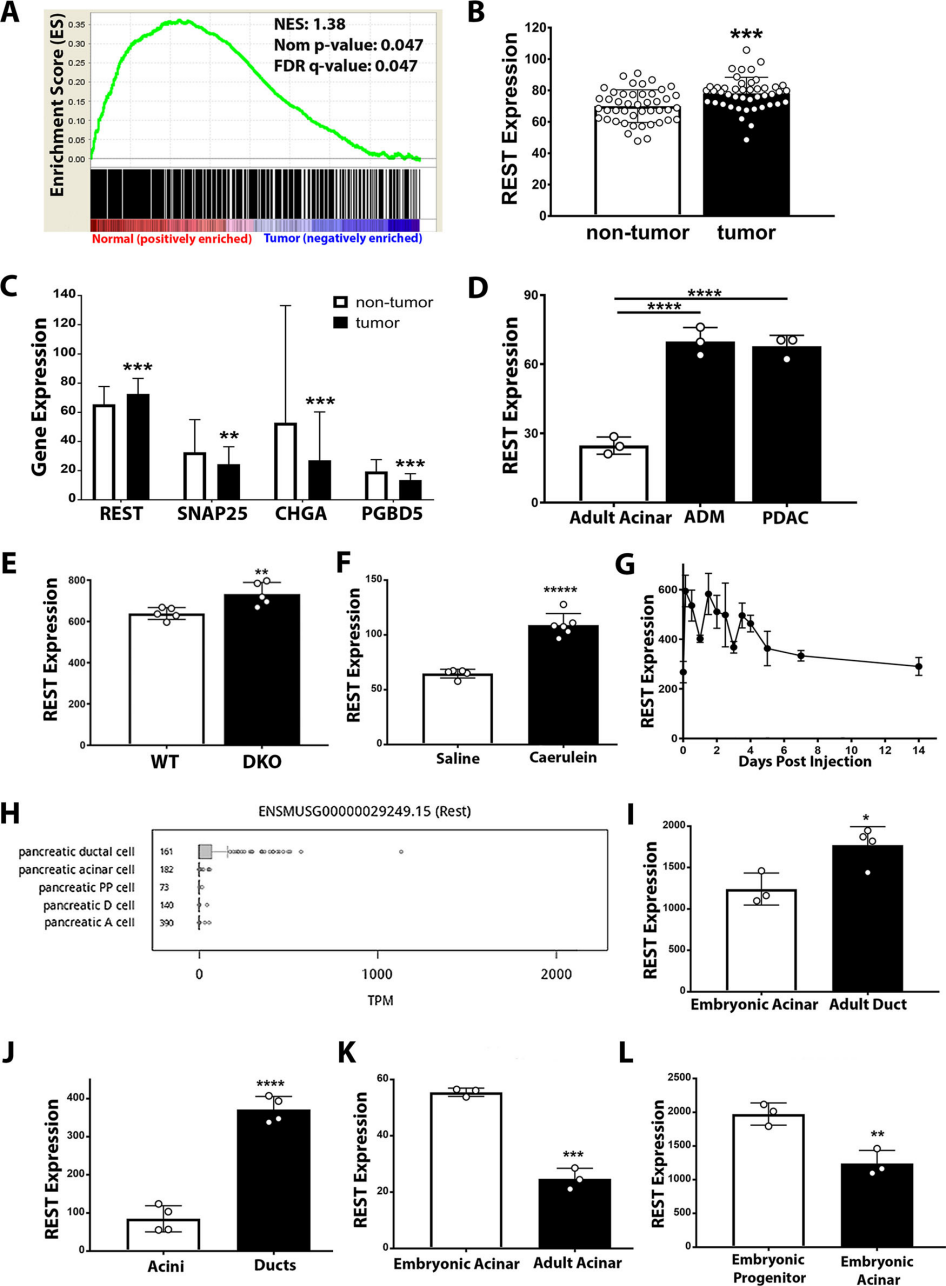
REST is differentially expressed during pancreatic injury and acinar differentiation. **a** Gene Set Enrichment Analysis of 971 REST target genes on eight normal human pancreas and 14 human PDAC samples (GSE71989). **b** REST gene expression values from dataset GSE28735 of non-tumor and tumor tissue from human pancreatic cancer patients (*n* = 45) (Avg ± SD). **c** Gene expression values of REST and REST target genes (Snap25, CHGA, PDGB5) from dataset GSE62452 of non-tumor and tumor tissue from human pancreatic cancer patients (*n* = 61–69) (Avg ± SD). **d** Rest gene expression values from dataset GSE116152 of wild-type adult mouse acinar cells, ADM cells, and tumor cells (*n* = 3) (Avg ± SD). **e** Rest gene expression values from dataset GSE44298 of wild-type mouse pancreas versus Mst1 and Mst2 KO mice (DKO) (*n* = 5) (Avg ± SD). **f** Rest gene expression values from dataset GSE109227 of wild-type mouse pancreas following caerulein treatments versus saline control (Avg ± SD). **g** Rest gene expression values from dataset GSE65146 of wild-type mouse pancreas following caerulein treatments. (Avg ± SD). **h** Boxplot of REST (ENSMUSG00000029249.15) expression level quartiles with outliers in FACS-sorted mouse pancreas cells. Graph from the USCS Genome Browser depicting the Tabula Muris single-cell RNA-seq dataset. Number of cells per cell type is shown. PP = pancreatic polypeptide, A = alpha, D = delta. **i** Rest gene expression values from dataset GSE54374 of E15.5 wild-type mouse acinar cells and 8–12-week ductal cells (Avg ± SD). **j** Rest gene expression values from dataset GSE70173 wild-type mouse acini or ductal organoids (Avg ± SD). **k** Rest gene expression values from dataset GSE116152 of wild-type mouse adult acinar cells and embryonic acinar cells (Avg ± SD). **l** Rest gene expression values from dataset GSE54374 of wild-type mouse pancreatic E15.5 acinar cells and E15.5 progenitor cells (Avg ± SD). *p < 0.05, **p < 0.001, ***p < 0.0001, ****p < 0.00001.

To investigate the role of REST in the exocrine pancreas, GSEA was performed on the gene expression profiles of normal human pancreas and pancreatic ductal adenocarcinoma tissues as described in the “Materials and methods” section. The REST target genes were highly enriched (i.e., upregulated in the normal pancreas with low *REST* expression) in both the large (Fig. 1a) and small REST target gene sets (Suppl Fig. 1A). In the GSEA that used 572 transcription factors and transcription factor binding sites as the gene set database, 57 of the 572 gene sets were upregulated in normal pancreas. While none of these 57 genes sets were significantly enriched (*p* < 0.05), the *REST* gene set was the top ranked (Suppl Table 2). These data suggest that REST activity is enhanced in human pancreatic ductal adenocarcinoma. Gene expression profiling from independent Gene Expression Omnibus (GEO) datasets^40,41^ confirms that *REST* mRNA levels are elevated in human pancreatic tumor samples (Fig. 1b,c) and correspond with lower levels of REST target genes such as *SNAP25*, *CHGA*, and *PDGB5* (Fig. 1c). Gene expression profiles from two independent datasets^42^ do not show significantly differential REST expression among stages of human PDAC (Suppl Fig. 1B,C), although the low number of available early and late human tumor samples makes analysis limited. Furthermore, survival of PDAC patients does not correlate with levels of *REST* expression (Suppl Fig. 1D).

The role of REST during the initiation of PDAC was then investigated. Gene expression profiling^43^ of a PDAC mouse model shows that *Rest* expression increases prior to cancer development and is elevated in ADM lesions relative to normal acinar tissue (Fig. 1d). Similarly, available profiling data from mice with pancreatic Mst1/2 knockout (DKO)^44^ that experience a loss of acinar mass and gain of ductal population also show an increase in REST expression compared with control (Fig. 1e). Pancreatitis is a known inducer of ADM in mice, so *Rest* gene expression was investigated from GEO datasets^45,46^ of caerulein-induced acute pancreatitis studies. *Rest* expression was increased in response to acute administration of caerulein (Fig. 1f, g) and decreased to baseline upon recovery (Fig. 1g). Of note, one GEO dataset^47^ of chronic mouse pancreatitis samples did not show increase of any *Rest* coding exons, but did show increased levels of the 3′UTR region (Suppl Fig. 1E). Overall, most independent datasets investigated suggest that Rest levels increased in response to injury and during ADM.

To better understand differences of *Rest* expression at the cellular level, the USCS Genome Browser was used to evaluate single-cell RNA-seq results from the Tabula Muris RNA-seq dataset^48–50^. Isolated pancreatic ductal cells showed greater levels of *Rest* expression compared with acinar and endocrine (pancreatic polypeptide, alpha, delta) pancreas cells (Fig. 1h). In agreement to this finding, independent GEO datasets^51,52^ that performed gene profiling of isolated mouse exocrine pancreas cells show greater *Rest* levels in adult ductal cells compared with embryonic acinar cells (Fig. 1i) or adult acinar cells (Fig. 1j). Deeper investigation of REST expression during mouse acinar maturation shows that adult acinar cells have less *Rest* expression than embryonic acini (Fig. 1k), and embryonic acini have lower *Rest* mRNA levels than embryonic progenitor pancreas cells (Fig. 1l), based on independent GEO datasets^43,51^. These data indicate that *Rest* expression decreases during acinar exocrine maturation and is maintained at lower levels than in duct cells.

### Development of a mouse model with conditional loss of REST in the exocrine pancreas

**Fig. 2:**
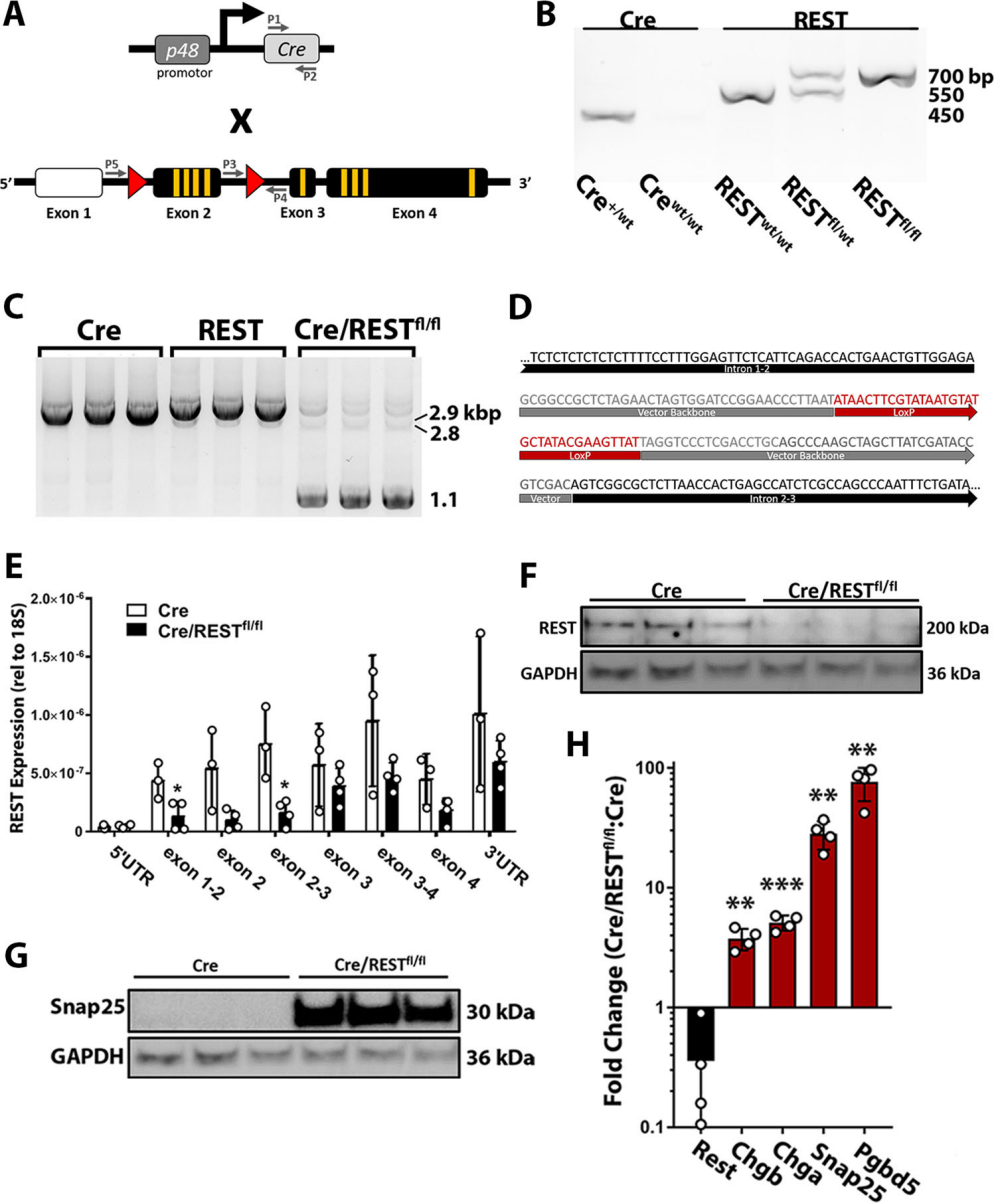
Transgenic mouse model with conditional loss of REST in the exocrine pancreas. **a** Schematic of REST knockout breeding. A breeder with Cre Recombinase gene downstream of the p48 promotor is crossed with a breeder containing LoxP sites (red triangles) flanking exon two of REST. White box indicates non-coding UTR. Yellow indicates zinc fingers. Gray “P” arrows indicate target region of PCR primers. **b** Gel electrophoresis of PCR products from genomic DNA for Cre Recombinase (P1 and P2) (~450 bps) and floxed *Rest* (P3 and P4) (WT: ~550 bps; Fl: ~700 bps). **c** Gel electrophoresis of PCR products from primers P5 and P4 encompassing LoxP sites of *Rest* exon two from pancreatic genomic DNA of 1–2-month-old mice. Wild-type product is ~2.8 kbs, floxed exon two product is ~2.9 kbs, and successfully excised exon two product is ~1.1 kb (*n* = 3) (Primer designs^30^). **d** Sanger Sequencing of PCR product (P4 and P5) from Cre/REST^fl/fl^ pancreatic DNA. Black indicates *Rest* introns, gray indicates original vector backbone, and red indicates LoxP sequence (*n* = 3). **e** qRT-PCR of *Rest* exon gene expression of 6-week-old mouse pancreas (normalized to 18S rRNA) (*n* = 3–4) (Avg ± SD). **f** Western blot of pancreatic protein with anti-Rest antibody (Proteintech) (MW = 200 kDa) (*n* = 3). **g** Western blot of pancreatic protein for Rest target gene Snap25 (MW = 30 kDa) (*n* = 3). **h** Gene expression of six-week-old Cre/RESTfl/fl pancreas relative to p48-Cre (normalized to 18S rRNA) (*n* = 4) (Avg ± SD). *p < 0.05, **p < 0.001, ***p < 0.0001.

To investigate the role of REST during acinar homeostasis and response to injury, a novel transgenic mouse model was developed with *Rest* conditionally knocked out in the exocrine pancreas. Mice with homozygous intronic *LoxP* sites flanking exon two of *Rest* (REST^fl/fl^)^30^ were crossed with mice expressing the Cre Recombinase transgene under the pancreas-specific p48 promotor (p48-Cre^+/−^) (Fig. 2a) to produce pancreas-specific REST knockout mice (Cre/REST^fl/fl^).

Mouse tail snips were genotyped to confirm the presence of the Cre Recombinase transgene (primers P1 & P2) and the homozygous *Rest LoxP* mutant allele (primers P3 & P4) (Fig. 2b). Genomic DNA from Cre/REST^fl/fl^ mouse pancreas showed a high Cre-mediated excision efficiency by PCR analysis of the targeted *Rest* exon two region (primers P5 & P4) (Fig. 2c). Sanger Sequencing of the PCR amplified exon two region from REST KO pancreatic DNA shows the loss of exon two between intron 1–2 and intron 2–3 (Fig. 2d). As expected, the presence of the targeting vector with the LoxP sequence was observed at the excision site, indicating a successful genomic KO event. Gene expression of *Rest* was quantified to confirm knockout. In accordance with a previous report using the REST^fl/fl^ model^11^, gene expression analysis showed loss of the targeted *Rest* exon two but expression of a truncated exon three and four *Rest* transcript (Fig. 2e). To evaluate loss of full-length REST protein in Cre/REST^fl/fl^ mice, western blot was performed on pancreatic lysate using five commercially available anti-REST antibodies and one custom-made antibody (Hsieh514^31^). While one antibody shows decrease in full-length REST protein (Fig. 2f), results among the other antibodies tested were variable (Suppl Fig. 2). Nevertheless, KO of *Rest* exon two was adequate to increase gene expression of known REST target genes such as *Chga, Chgb, Snap25*, and *Pgbd5*^16,39^ in the pancreas and increase protein of Snap25 (Fig. 2g, h), suggesting successful abrogation of REST function as a transcriptional repressor.

### REST knockout increases production of zymogen granules in pancreatic acini

**Fig. 3:**
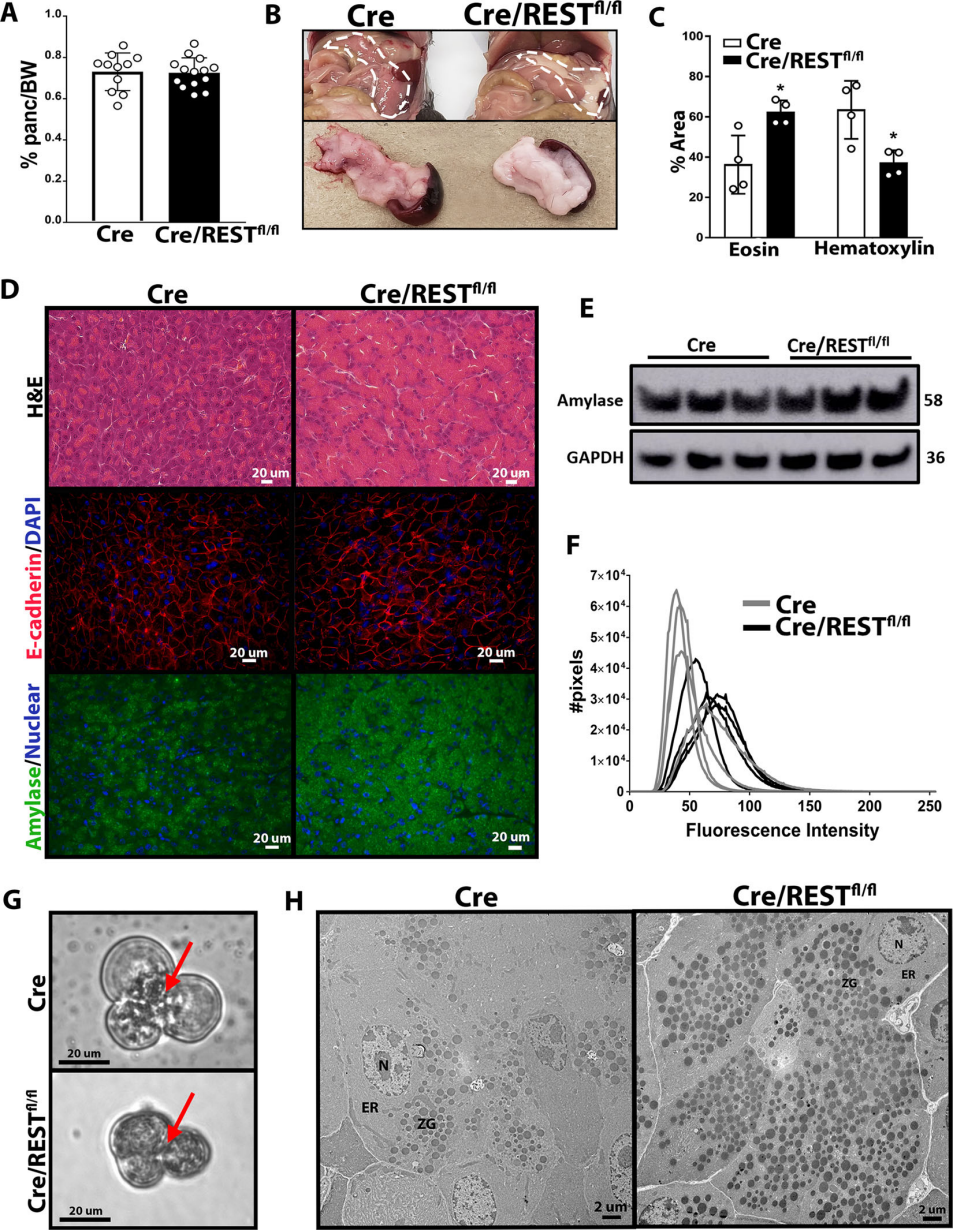
Phenotype of pancreata from REST Knockout mice. **a** Pancreas weight normalized by body weight of individual Cre and Cre/REST^fl/fl^ mice (*n* = 11–14) (Avg ± SD). **b** Representative images of Cre and Cre/REST^fl/fl^ pancreas from abdomen and excised spleen. **c** Percent of total tissue area stained purple versus pink by H&E stain, respectively, of Cre and Cre/REST^fl/fl^ pancreas (*n* = 4) (Avg ± SD). **d** Representative images of stained pancreas tissue of 2-month-old Cre/REST^fl/fl^ and control. Staines include H&E, immunofluorescence against E-cadherin (red) and nucleus (blue), and immunofluorescence against amylase (green) and nucleus (blue) (*n* = 4). **e** Western Blot of pancreatic protein for amylase (MW = 58) (*n* = 3). **f** Histogram of immunofluorescence intensity after amylase staining (*n* = 4). **g** Brightfield imaging (40×) of acinar clusters from 6-week-old Cre/REST^fl/fl^ and control mice. Red arrows indicate apical surface of acini cluster. **h** Transmission electron microscopy of 6-week-old Cre and Cre/REST^fl/fl^ pancreas (400×). N = nucleus, ER = endoplasmic reticulum, ZG = zymogen granules.

Mice devoid of pancreatic REST had the same pancreatic weight normalized to body weight as control mice (Fig. 3a). Interestingly, the gross appearance of the Cre/REST^fl/fl^ mouse pancreas appeared whiter in color, distended, and fewer separations between lobules compared with control mice (Fig. 3b). Histologic observation of pancreatic acini showed increased eosinophilic staining of the cytoplasm from Cre/REST^fl/fl^ mice (Fig. 3c, d), suggesting an increase in intracellular protein content. Pancreas from REST KO mice displayed atypia and acinar cell swelling, observable by e-cadherin staining of acinar cell boundaries (Fig. 3d). Although Cre/REST^fl/fl^ mice do not appear to have greater total amylase levels (Fig. 3e), immunofluorescence staining suggests loss of REST disrupts the cytosolic distribution of amylase within the acinar cell (Fig. 3d, f). Of note, pancreatic endocrine function was euglycemic in REST KO mice, as evaluated by fasted blood glucose levels and glucose tolerance testing in comparison to control mice (Suppl Fig. 3A-C).

At the cellular level, brightfield microscopic images of REST KO acinar clusters from digested mouse pancreas showed aberrant dark granule localization from the apical to the basal surface of the acinar cluster (Fig. 3g). Transmission electron microscopy of Cre/REST^fl/fl^ and Cre control tissue revealed that REST KO pancreata had a significant increase in intracellular zymogen granules with most cells essentially filled with granules versus the apical accumulation in the Cre mice (Fig. 3h).

### Loss of REST in pancreatic acini inhibits ductal transdifferentiation in vitro

**Fig. 4:**
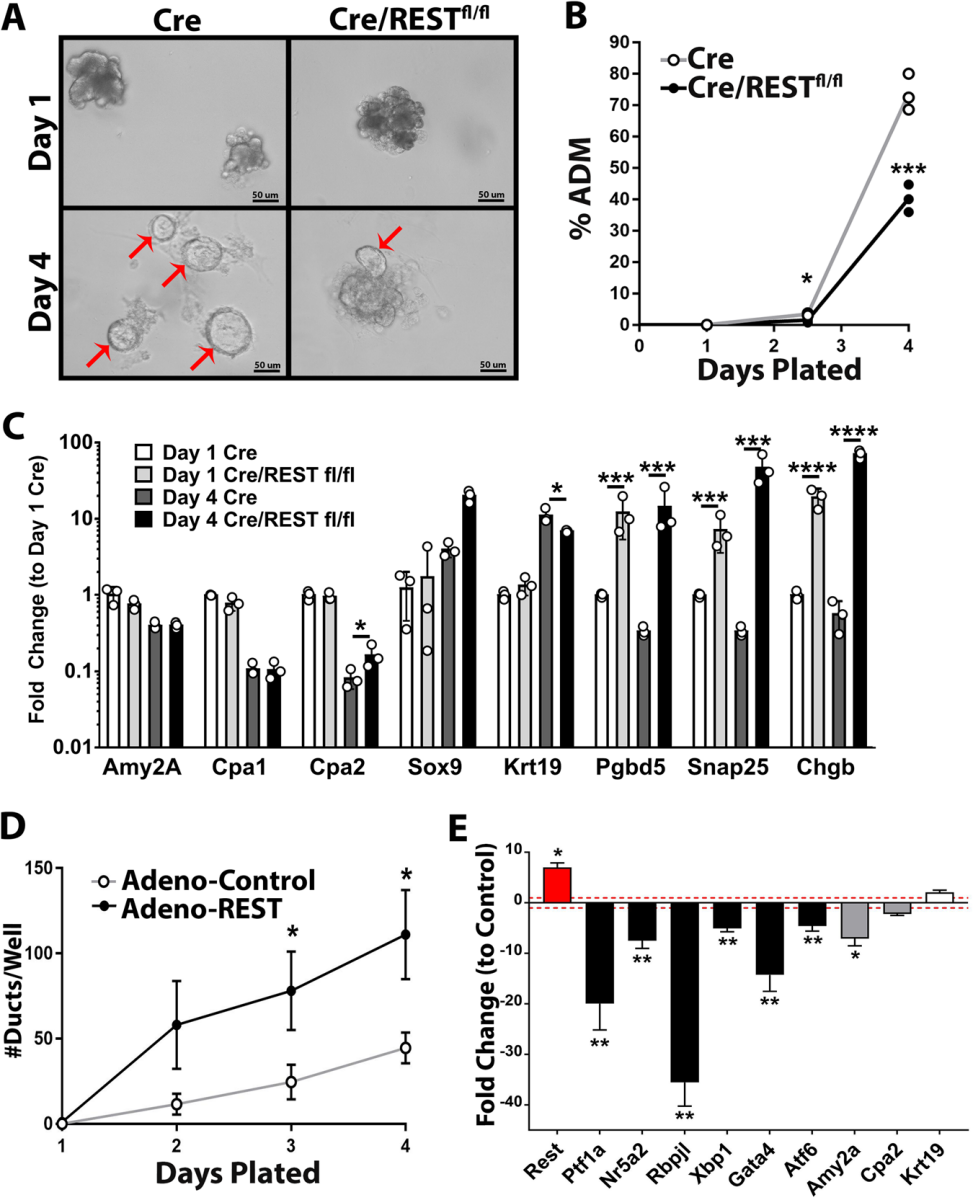
Loss of REST in pancreatic acini inhibits ductular transdifferentiation during in vitro ADM. Primary mouse pancreatic acini from Cre/REST^fl/fl^ or control Cre mice were cultured as organoids on Matrigel over 4 days. **a** Representative brightfield images (40×) of acinar and ductal organoids at day 1 and day 4. Red arrows indicate ductal-like structures with hollow lumen. **b** Percent of acinar clusters that formed ductal-like structures when cultured on Matrigel (Avg ± SD). **c** Fold changes in gene expression of acinar genes (*Amy2a, Cpa1, Cpa2*), progenitor gene (*Sox9*), ductal gene (*Krt19*), and Rest target genes (*Pgbd5, Snap25, Chgb*) relative to Cre acini at day 1 (normalized to 18S rRNA) (*n* = 3) (Avg ± SD). **d** Number of ducts per well of adenovirus-REST transfected and control adenovirus-transfected wild-type mouse acini in Matrigel (*n* = 4) (Avg ± SD). **e** Fold change at day 4 in gene expression of adenovirus-REST transfected relative to control adenovirus-transfected WT mouse acini. Red dotted line at fold change = 1 (normalized to 18S rRNA) (*n* = 4) (Avg ± SEM). *p < 0.05, **p < 0.01, ***p < 0.0001, ****p < 0.00001.

To investigate the capability of acinar cells to transdifferentiate into ductal-like cells in the absence of REST, acini from Cre/REST^fl/fl^ mice were cultured as organoids and induced to undergo ADM by plating in Matrigel. Mouse acini with loss of REST produced fewer and smaller ductal morphologies relative to control pancreas over 4 days of culture (Fig. 4a, b). As ADM is a stepwise succession of acinar de-differentiation then progenitor transdifferentiation into ductal-like identity, gene expression was investigated to determine molecular differences in pancreatic identity. Acinar markers *Amy2a*, *Cpa1*, and *Cpa2* decreased in both groups over the course of 4 days, but REST KO cells show less decrease in acinar marker *Cpa2* compared with control cells (Fig. 4c). Congruously, REST KO cells showed a greater increase in progenitor marker *Sox9* at day 4 and a lower increase in ductal marker *Krt19* at day 4 compared with control cells (Fig. 4c). Model validation is supported by increased REST target gene expression (*Pgbd5, Snap25, Chgb*) in Cre/REST^fl/fl^ cells compared with control (Fig. 4c). Together, these data imply that REST is important for pancreatic acini to properly transdifferentiate into ductal cells in response to induced transdifferentiation.

The inverse relationship was observed when REST is overexpressed in wild-type mouse acini and ADM is induced. Adenoviral-REST was transfected into wild-type pancreatic acini that were subjected to organoid culturing over the course of 4 days. Acini with elevated levels of *Rest* produced more ducts per well compared with adenoviral-control (Fig. 4d). Furthermore, the seven-fold increase in *Rest* consequently doubled the expression of ductal identity marker *Krt19* and dramatically decreased expression of acinar identity markers (*Amy2a, Cpa2*) and acinar transcription factors (*Ptf1a, Nr5a2, Rbpjl, Xbp1, Gata4, Atf6*) relative to control (Fig. 4e). These data suggest that REST promotes ductal-like transformation of acinar cells.

### Loss of Rest in pancreatic acini increases caerulein-induced acute pancreatitis

**Fig. 5:**
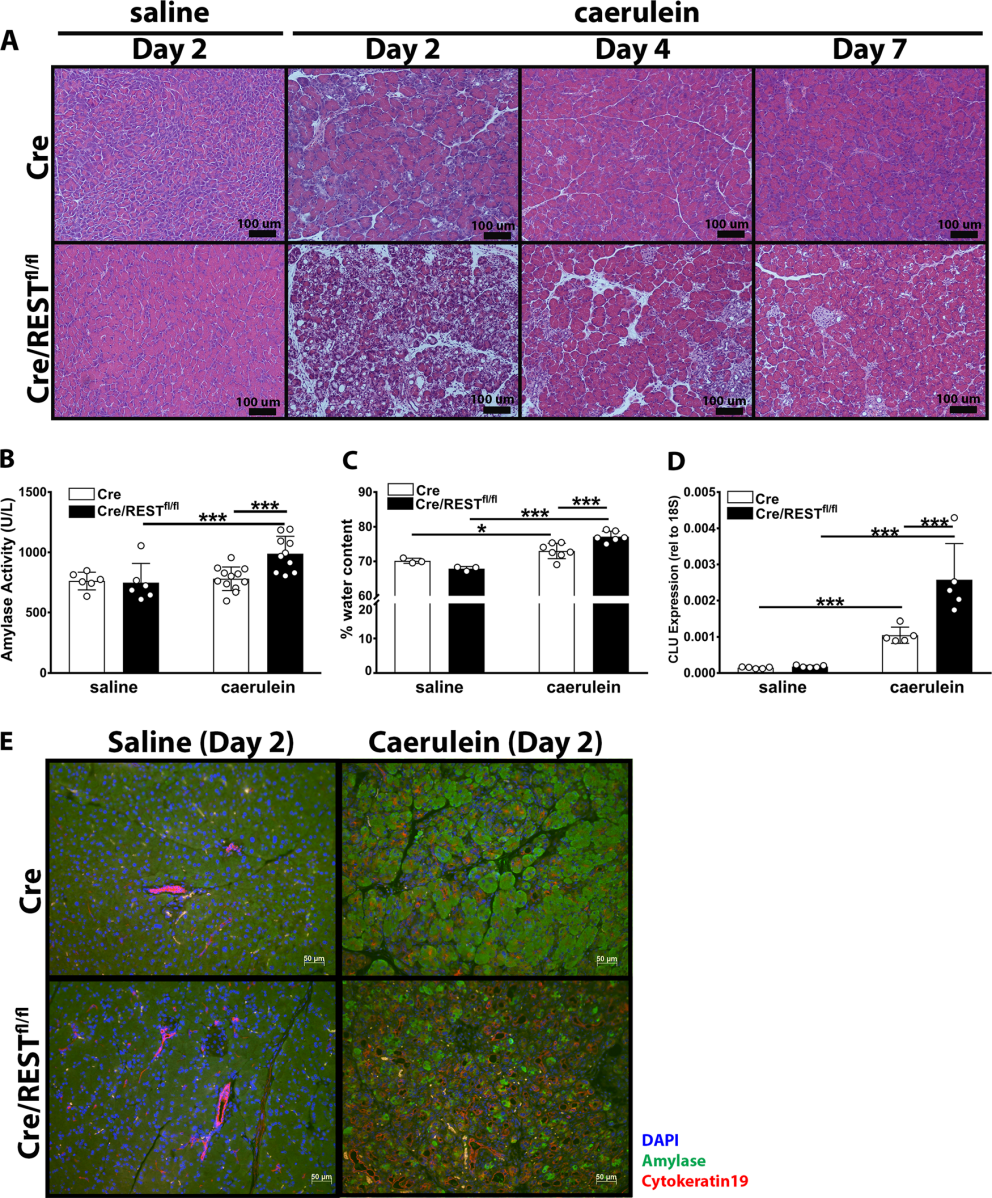
Caerulein-induced acute pancreatitis is accelerated in REST KO mice. **a** Representative H&E stained slides of saline and caerulein treated mice at 2, 4, and 7 days following injections. **b** Blood serum amylase activity 2 days after caerulein or saline injection (*n* = 6–12) (Avg ± SEM). **c** Edema presented as percent water content of pancreas from caerulein-induced acute pancreatitis at day 2 post-injections (*n* = 3–7) (Avg ± SD). **d** *Clusterin* gene expression of mouse pancreas 2 days after injections of saline or caerulein (normalized to 18S rRNA) (*n* = 5) (Avg ± SD). **e** Representative immunofluorescence images of mouse pancreas at day 2 post-injections stained against amylase (green), cytokeratin19 (red), and DAPI (blue) (*n* = 4). *p < 0.05, **p < 0.001, ***p < 0.0001, ****p < 0.00001.

Aberrant upregulation of zymogen granules during homeostatic conditions suggests that REST knockout mice would be more sensitive to autodigestion-related injury. Furthermore, if REST is required for acinar transdifferentiation into a refractory duct-like state as a protective mechanism, then loss of REST in acinar cells would lead to greater injury severity. To test the effects of REST knockout on acinar cells during tissue damage and recovery, mice were induced with acute pancreatitis using elevated doses of caerulein. Histopathology revealed REST KO mice had greater tissue injury (Fig. 5a). Microscopic scoring showed trending but not statistical increases in four tissue parameters scored at day 2 in REST KO mice (Suppl Fig. 4A-E). Sensitive biochemical methods were used to quantify the differences in pancreatitis severity at the day 2 of peak injury. Serum amylase levels were higher in REST knockout mice compared with controls (Fig. 5b). Furthermore, REST knockout mice had greater pancreatic edema in response to caerulein-induced injuries (Fig. 5c). Cre/REST^fl/fl^ had greater gene expression of clusterin (Fig. 5d), a molecular chaperone that protects against acinar cell stress^53^. Acute caerulein treatment was sufficient to induce an increase in the cytokeratin19^+^ ductal population and decrease in the amylase^+^ acinar population (Fig. 5e). These data show REST knockout mice are more sensitive to caerulein-induced acini overstimulation and transdifferentiation, suggesting REST plays a protective role against pancreatic tissue damage.

## Discussion

Since plasticity serves to protect cells from injury, a deeper molecular understanding of pancreas cell differentiation is a major focus for studies on regeneration and replacement therapies of both exocrine and endocrine cell types post tissue damage. As a master negative regulator of neurogenesis, REST prevents precocious expression of neuronal genes and has been shown as an important regulator of pancreas neuroendocrine identity^7^. However, beyond neuronal gene repression, little is known about the role of REST within exocrine cell identity where it is normally highly expressed. Analysis using independent Gene Expression Omnibus datasets and GSEA analysis of human and mouse samples showed increased levels of *Rest* in response to injuries such as inflammation, aberrant plasticity, and tumor development. To elucidate the role of REST elevation upon insult, cell-type-specific expression was analyzed and revealed differential levels of *Rest* expression within exocrine cell types (acinar vs ductal) and within states of acinar maturity during development. From these findings, we demonstrate the regulation of acinar cellular homeostasis by REST and its protective role in pancreatic exocrine cells against injury.

To investigate REST activity in the exocrine pancreas, we developed and characterized a conditional REST KO model. By crossing REST^floxP/floxP^ mice^30^ with mice expressing Cre from the p48/Ptf1a promotor, exon two containing the coding sequence for the first four zinc fingers of *Rest* was excised, thereby knocking out REST translation and the N-terminal activity region. The mouse KO model was validated at the genomic, proteomic, and functional level by DNA sequencing, western blot, and qPCR of REST target genes, respectively. The variable results presented of REST western blot analysis could be due to numerous technical challenges. Quantifying a decrease in the already low endogenous levels of full-length REST in acinar cells would require high antibody sensitivity. Others have reported the insensitivity and lack of specificity of REST antibodies^54^, and different REST isoforms and glycosylation states have caused discrepancies of reported REST molecular weight bands in the literature^55^. Of note, this model creates REST KO at the germline level; therefore, all pancreas precursors may experience an excision event during the progenitor stage of p48/Ptf1a expression. Even so, the p48-Cre/REST^fl/fl^ mouse is an appropriate model for KO of exocrine REST because (1) p48/Ptf1a expression is maintained in differentiated acinar cells and (2) REST levels are endogenously low in endocrine cells^13^.

One phenotypic consequence from a loss of REST in acinar cells was an increase of zymogen granules. These organelle packages contain digestive enzymes and are produced at the endoplasmic reticulum before being translocated to the apical surface of the acinar cell for secretion. REST KO acinar cells showed cellular swelling and an elevated concentration of zymogen granules, likely causing the observed improper localization into the basal surface. This phenotype is consistent with other cell types that show loss of REST increased dense-core vesicle gliosecretion^56^, and increase of REST caused a decrease in secretory granules^57^. The accumulation of granules in REST KO acini is likely from increased production rather than lack of secretion since loss of REST activity increases target gene expression, several of which are players in exocytosis machinery^58^. We postulate the observed elevated neuronal properties of the exocrine pancreas in REST knockout mice increased sensitivity to neuronal stimulation of zymogen granule production.

To determine the role of REST in regulating acinar identity, acinar clusters from mice were stimulated ex vivo to undergo ADM. In the absence of REST, acinar cells demonstrated an impeded ability to transdifferentiate into ductal-like cells. Inversely, REST overexpression within acinar cells showed a heightened ductular transformation. These data suggest that the observed increased levels of REST expression in ductal cells relative to acinar cells is important in promoting ADM. Elevated levels of REST correlate with greater pluripotency properties (reviewed in refs. ^38,59^); therefore, increased *REST* expression may promote the progenitor potential of the ductal precursor state during ADM.

Acinar cell plasticity into a ductal-like identity is a critical protective mechanism against common pancreas injuries such as inflammation^60^. Transient ADM promotes resistance to cellular exocrine damage and allows regeneration of the lost acinar population once the injury is resolved. To ascertain the importance of REST expression during exocrine damage and recovery, REST KO mice were subjected to inflammation-related injury by acute injections of caerulein. Mice with loss of REST displayed greater serum amylase levels and edema, which could be explained by enhanced sensitivity to caerulein overstimulation and/or lost regulation of acinar homeostasis. Importantly, pancreatitis-damaged tissue from both REST KO and Cre control mice demonstrated a loss of acinar cells and a gain of ductal cells. While the proposed role of REST in promoting ADM would have predicted fewer ADM-like lesions in REST KO mice, other parameters within this model must be taken into consideration, such as the influences of neuronal, immune, and extracellular matrix components. Furthermore, loss of REST only partially impaired in vitro ADM, suggesting REST plays an important but not critical role for acinar transdifferentiation. The severe degree of injury in REST KO mice during pancreatitis could overpower the dependence on REST during induced ADM through an alternative mechanism. Taken together, these data suggest a protective role of REST against exocrine injury and maintaining pancreas stability.

Regulation of cell identity has a broad range of therapeutic applications to common pancreas-related clinical morbidities such as diabetes, pancreatitis, and pancreatic cancer development. This study exposed a previously unknown role of REST in progenitor differentiation, specifically within the exocrine compartment of the pancreas. REST was shown as a critical regulator of pancreas homeostasis that protects the pancreatic acini from injury by promoting transdifferentiation into ductular cells. Future directions could investigate REST in maintaining acinar cell organization during KRAS-driven ADM, which is believed to be an initial event towards pancreatic cancer development^26^. Elucidating the molecular mechanism of pancreas cell differentiation will uncover novel therapeutic targets to exploit the innate plasticity for treatment options.

## Supplementary information

Supplemental Figure Legends

Supplemental Figure 1

Supplemental Figure 2

Supplemental Figure 3

Supplemental Figure 4

Supplemental Table 1

Supplemental Table 2
