# Supplementary material for: Loss of RE-1 silencing transcription factor accelerates exocrine damage from pancreatic injury
Source: Cell Death Dis. 2020 Feb 20;11(2):138. doi: 10.1038/s41419-020-2269-7 (PMC7033132; doi:10.1038/s41419-020-2269-7)
Supplement: Supplementary file 1 — Supplemental Figure Legends [file 41419_2020_2269_MOESM1_ESM.docx]

Supplemental figure legends for manuscript

**loss of RE-1 Silencing Transcription Factor accelerates EXOCRINE damage from pancreatic injury**

**Julie K. Bray^1^, Ola A. Elgamal^2^, Jinmai Jiang^2^, Lais S. Wright^2^, Dhruvitkumar S. Sutaria^2^, Mohamed Badawi^3^, Madeline G. Borcyk^2^, Xiuli Liu^1^, Kristianna M. Fredenburg^1^, Martha L. Campbell-Thompson^1^ and Thomas D. Schmittgen^2,*^**

^1^Department of Pathology, Immunology, and Laboratory Medicine, College of Medicine, University of Florida ^2^Department of Pharmaceutics, College of Pharmacy, University of Florida

^3^Division of Pharmaceutics, College of Pharmacy, Ohio State University

**Supplemental Figure 1: REST is differentially expressed during pancreatic injury.** (A) Gene Set Enrichment Analysis report of 24 REST target genes on eight normal human pancreas and fourteen human PDAC samples (GSE71989). (B) REST expression values from TCGA PAAD dataset. Statistical significance analyzed with Kruskal-Wallis test (p=0.1610) (n=3-128) (Avg ± SD). (C) REST gene expression values from dataset GSE26452 of human PDAC tissue. Statistical significance analyzed with Kruskal-Wallis test (p=0.0591) (n=3-46) (Avg ± SD). (D) Kaplan Meier survival analysis of PDAC patients according to REST expression from TCGA PAAD dataset (n=73). (Avg ± SD). (E) Rest gene expression values from dataset GSE41418 of mouse pancreas after caerulein-induced chronic pancreatitis (n=6). Genome probes 1425564_at, 1425565_at, and 1425565_at target coding regions and probe 1428227_at targets the 3’ UTR region of Rest. (Avg ± SD). ****p<0.00001.

**Supplemental Figure 2: REST protein levels in REST KO mouse pancreas.** Western blot of whole pancreas protein from one to two-month-old mice using four commercially available anti-REST antibodies: (A) ab202962-reported 122 kDa, then re-probed with (B) Hsieh514- reported ~200 kDa). (C) Aviva, ARP32086_P050-reported 120 kDa. (D) Millipore 07-579-reported 200 kDa. (E) ab21635-reported 121 kDa. M= Marker.

**Supplemental Figure 3: Characterization of mouse pancreas endocrine function in response to REST KO**. (A) Blood glucose levels of mice faster 12 hours overnight. Data shown as males only (n=5-6), females only (n=7-9), or combined (n=12-15) (Avg ± SD). (B) Blood glucose levels of mice fasted (twelve hours) then injected with 1-2 mg glucose/g body weight. Glucose measurements were taken at increments of 30 minutes post-injection for up to 120 minutes. Area under the glucose curve quantified in (C). Data shown as males only (n=7-8), females only (n=7), or combined (n=14-15) (Avg ± SD).

**Supplemental Figure 4: Quantification of histological injury of caerulein-induced acute pancreatitis.** Blinded pathologist scoring of H&E staining of pancreas tissue slides from Cre and Cre/REST^fl/fl^ mice injected with saline (control) (n=1) or caerulein (n=5) for two days. Experimental groups are saline (day 2 post-injections) and caerulein treated (2, 4, and 7 days post- injections). (A) Total Necrosis Score, (B) Total Inflammation Score, (C) Edema Score, (D) Atrophy score, (E) Total histology score, as a product of total necrosis, inflammation, and edema score. (n=2-5) (Avg ± SD).
