# Supplementary figures and images for: Loss of RE-1 silencing transcription factor accelerates exocrine damage from pancreatic injury
Source: Cell Death Dis. 2020 Feb 20;11(2):138. doi: 10.1038/s41419-020-2269-7 (PMC7033132; doi:10.1038/s41419-020-2269-7)

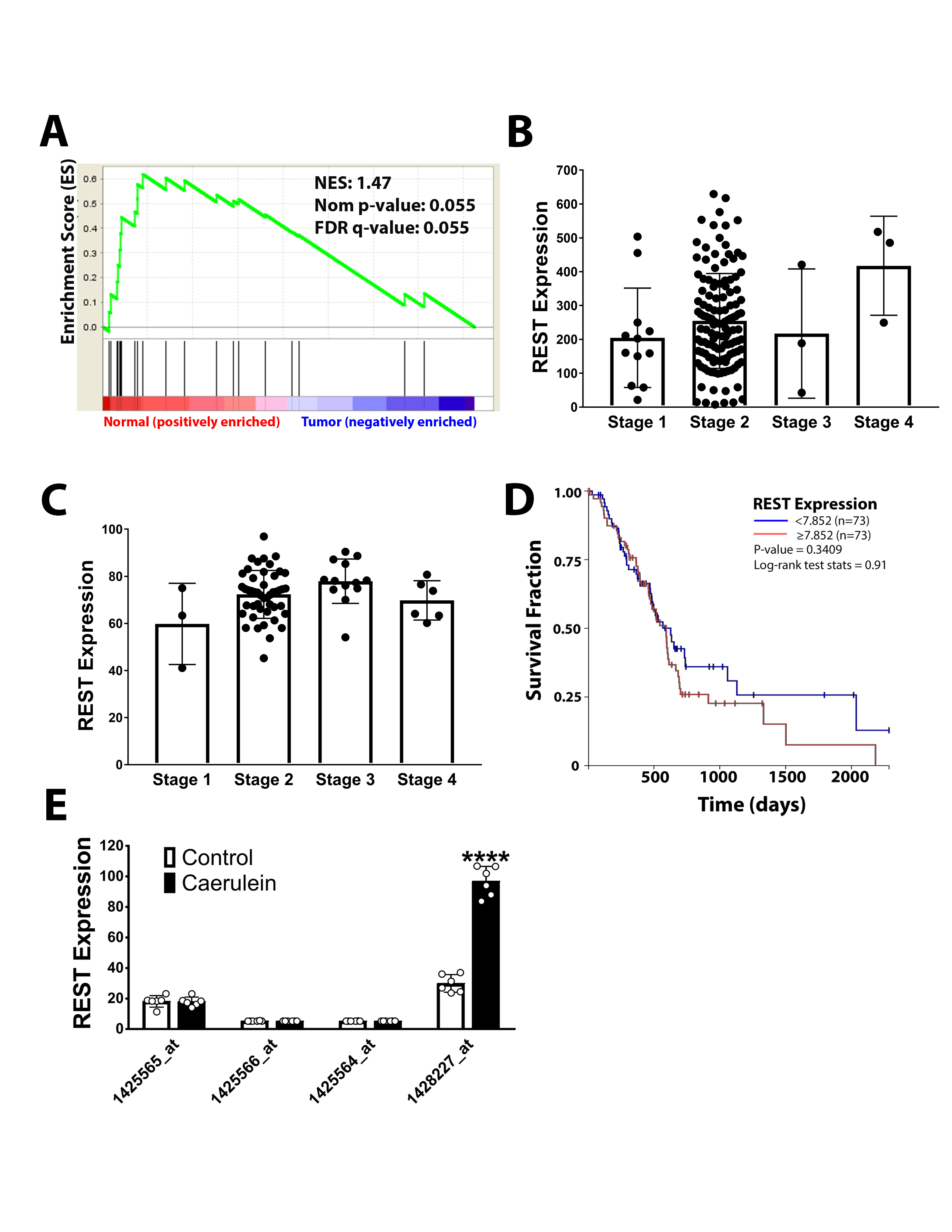

Supplement: Supplementary file 2 — Supplemental Figure 1 [file 41419_2020_2269_MOESM2_ESM.tif]

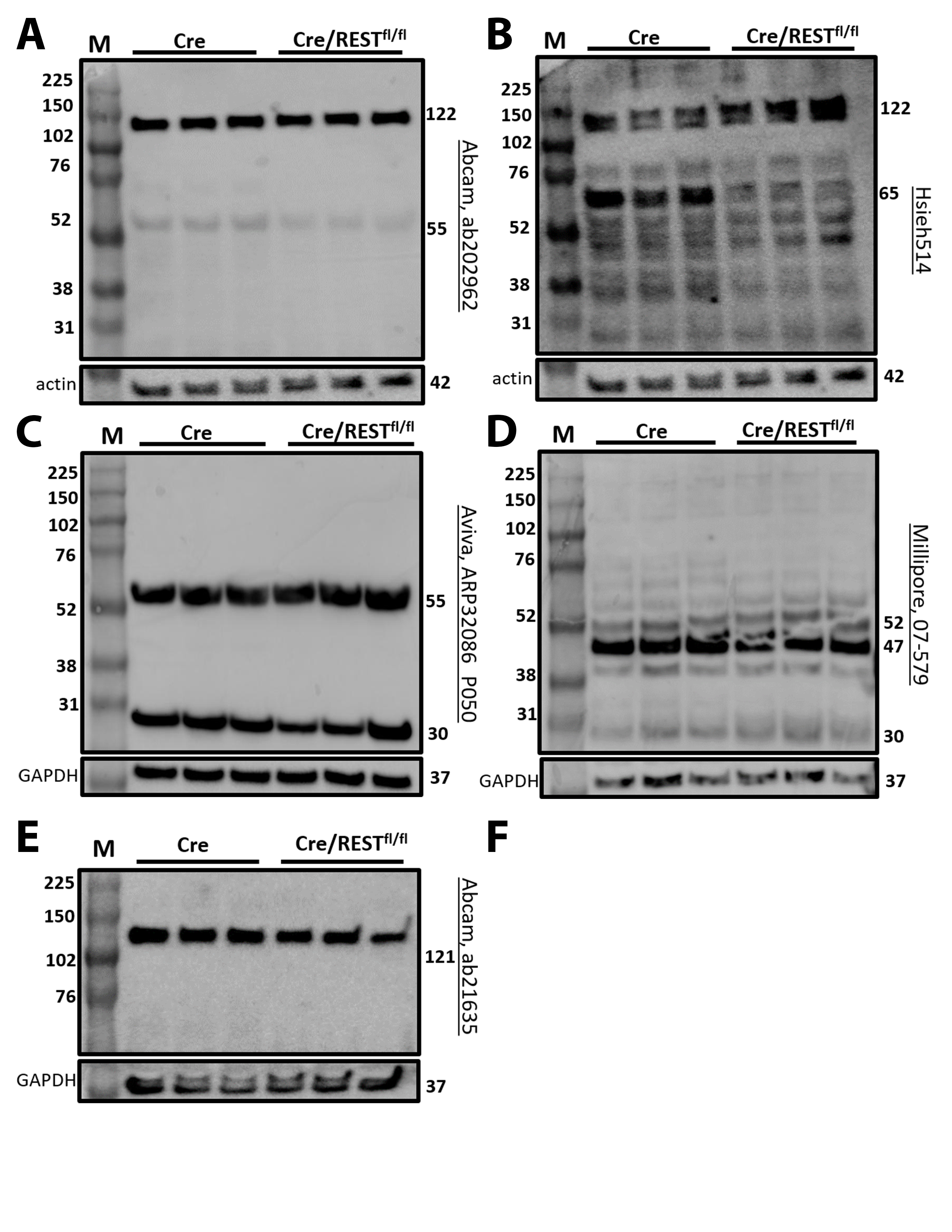

Supplement: Supplementary file 3 — Supplemental Figure 2 [file 41419_2020_2269_MOESM3_ESM.tif]

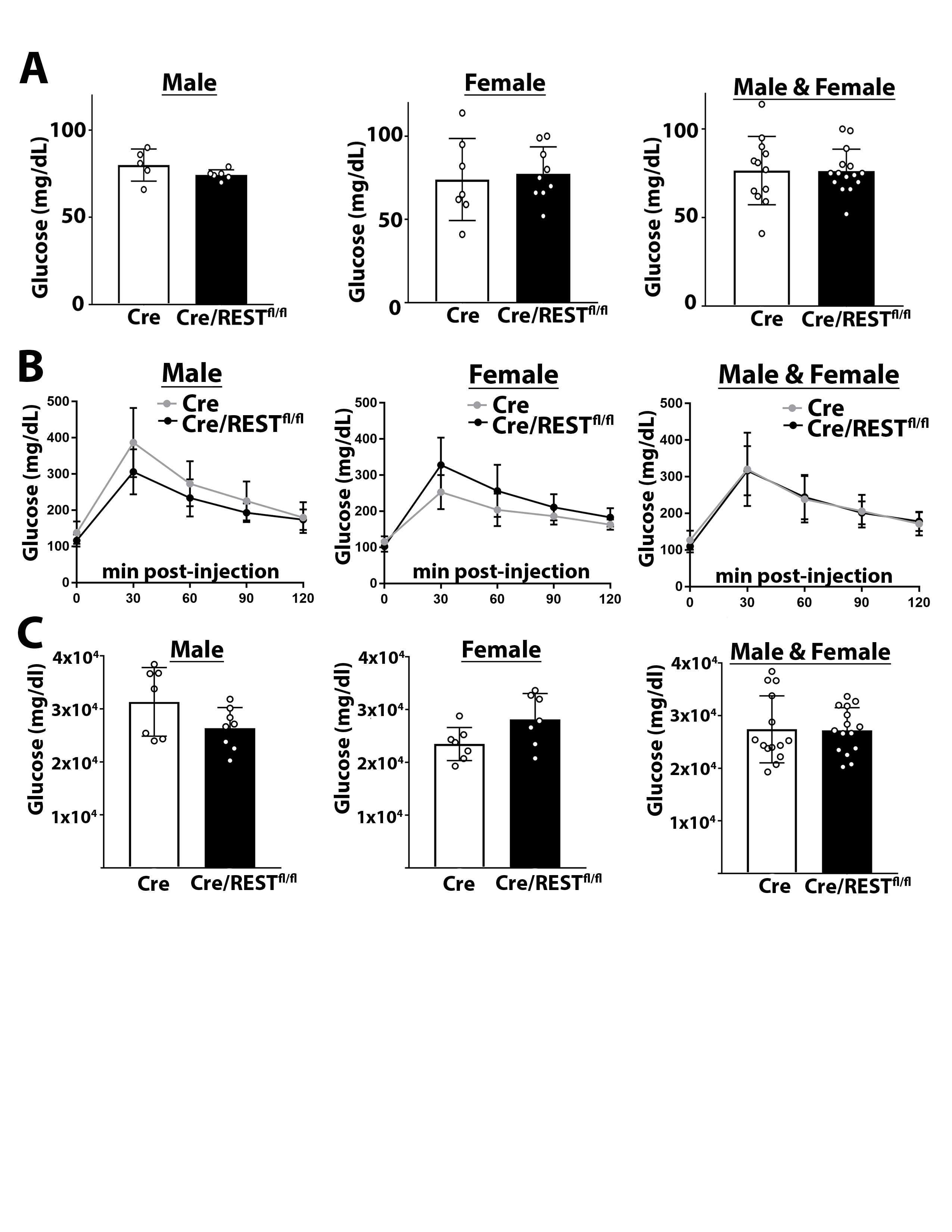

Supplement: Supplementary file 4 — Supplemental Figure 3 [file 41419_2020_2269_MOESM4_ESM.tif]

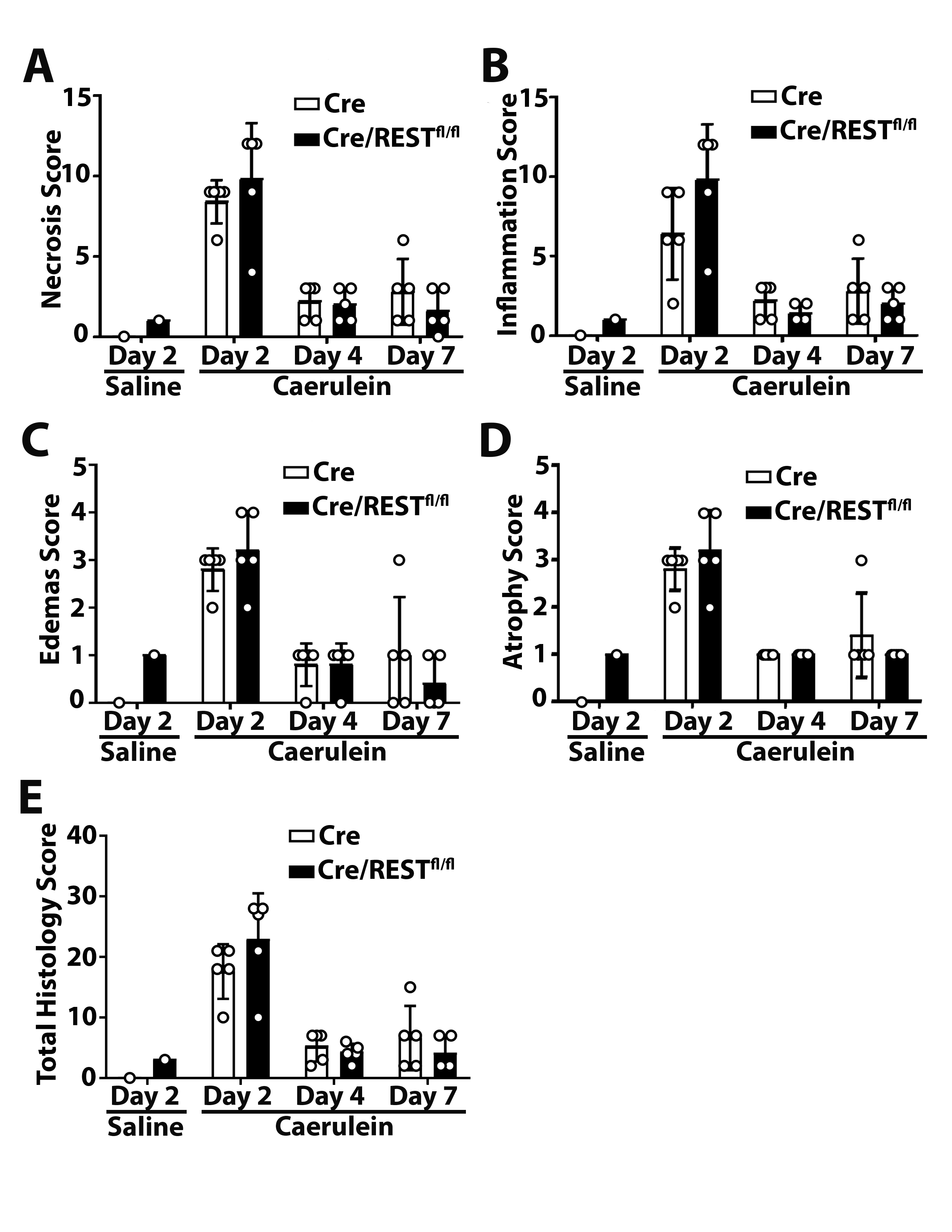

Supplement: Supplementary file 5 — Supplemental Figure 4 [file 41419_2020_2269_MOESM5_ESM.tif]
